# Advances in Anti-Cancer Immunotherapy: Car-T Cell, Checkpoint Inhibitors, Dendritic Cell Vaccines, and Oncolytic Viruses, and Emerging Cellular and Molecular Targets

**DOI:** 10.3390/cancers12071826

**Published:** 2020-07-07

**Authors:** Emilie Alard, Aura-Bianca Butnariu, Marta Grillo, Charlotte Kirkham, Dmitry Aleksandrovich Zinovkin, Louise Newnham, Jenna Macciochi, Md Zahidul Islam Pranjol

**Affiliations:** 1 School of Life Sciences, University of Sussex, Falmer, Brighton BN1 9QG, UK; e.alard@sussex.ac.uk (E.A.); ab923@sussex.ac.uk (A.-B.B.); mg473@sussex.ac.uk (M.G.); clk29@sussex.ac.uk (C.K.); l.j.newnham@sussex.ac.uk (L.N.); j.macciochi@sussex.ac.uk (J.M.); 2 Department of Pathology, Gomel State Medical University, 246000 Gomel Region, Belarus; zinovkin2012@gmail.com

**Keywords:** CAR-T cell, checkpoint inhibitor, dendritic cell vaccines, oncolytic viruses, tumour-induced immune evasion, immunosuppression, drug resistance, galectin-1, cathepsin D, OX40

## Abstract

Unlike traditional cancer therapies, such as surgery, radiation and chemotherapy that are typically non-specific, cancer immunotherapy harnesses the high specificity of a patient’s own immune system to selectively kill cancer cells. The immune system is the body’s main cancer surveillance system, but cancers may evade destruction thanks to various immune-suppressing mechanisms. We therefore need to deploy various immunotherapy-based strategies to help bolster the anti-tumour immune responses. These include engineering T cells to express chimeric antigen receptors (CARs) to specifically recognise tumour neoantigens, inactivating immune checkpoints, oncolytic viruses and dendritic cell (DC) vaccines, which have all shown clinical benefit in certain cancers. However, treatment efficacy remains poor due to drug-induced adverse events and immunosuppressive tendencies of the tumour microenvironment. Recent preclinical studies have unveiled novel therapies such as anti-cathepsin antibodies, galectin-1 blockade and anti-OX40 agonistic antibodies, which may be utilised as adjuvant therapies to modulate the tumour microenvironment and permit more ferocious anti-tumour immune response.

## 1. Introduction

Cancer remains a main global challenge due to the lack of early diagnosis, the inherent biological complexity of the tumour microenvironment (TME) and the unavailability of highly efficacious treatment strategies. In year 2015 to 2017, there were 367,167 new cases of cancers, with 165,000 deaths in the UK alone [1], indicating an urgent need for new effective treatment plans in place. Novel approaches developed to reduce tumour resistance to chemo- and radiotherapy and a combination of these have certainly improved treatment efficacy, however, overall clinical outcome still remains poor due to drug resistance and adverse events [2]. Thus, in recent years, utilising a host’s immunity to control and eliminate cancer has been developed which underpins the principles of anti-cancer immunotherapy.

The concept of immunotherapy shifts the focus of “targeting” from tumour itself to a more personalised approach, where the host’s immune system is programmed to directly or indirectly attack cancer cells. Several of these therapeutic strategies have been developed and approved for clinics, however, due to low treatment efficacy, drug resistance and tumour-induced immunosuppression, investigations are ongoing in the development of new adjuvant treatments and optimisation of existing immunotherapies, reviewed elsewhere [3,4]. Although promising outcomes have been observed in patients with haematological malignancies, there are numerous obstacles within the TME of solid tumours which pose many challenges in achieving clinical benefits, especially in patients with advanced stage cancers (discussed below). Therefore, in this review, we provide an overview of the latest developments of selected immunotherapy strategies such as chimeric antigen receptor (CAR) T cell, checkpoint inhibition, dendritic cell (DC) vaccines, and oncolytic viruses (OV) in both haematological cancers and solid tumours. We also explore recent advancements of newly discovered pro-tumourigenic and immunosuppressive candidates and their potential targeting in anti-cancer immunotherapies.

## 2. The Chimeric Antigen Receptor (CAR) T Cell

**Figure 1:**
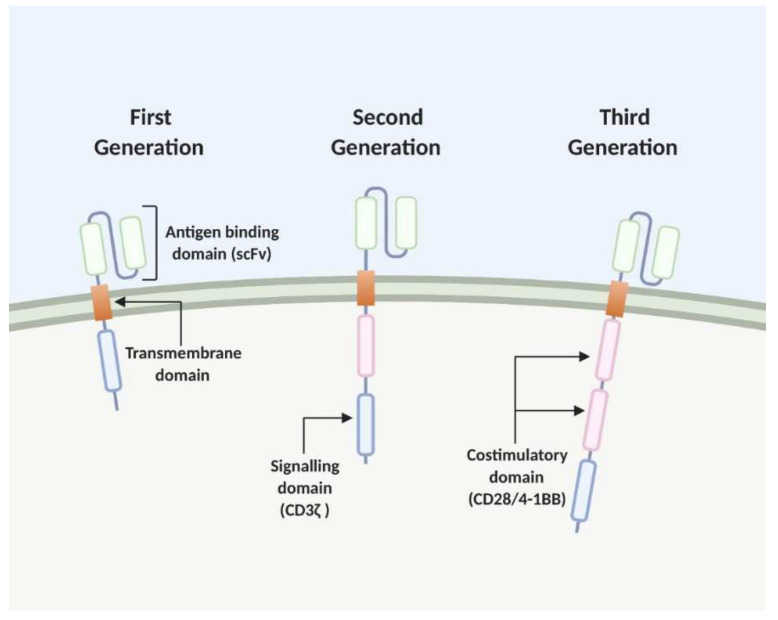
Chimeric antigen receptor structure. CARs follow a generic design of four components. First generation CARs are composed of an extracellular antigen binding domain synthesised from the variable heavy and light chains of mAbs (scFv), a transmembrane domain and a CD3ζ intracellular signalling domain. Second generation CARs have an additional co-stimulatory domain, typically CD28 or 4-1BB, and third generation CARs will have two of these costimulatory domains.

The adoptive transfer of CAR T cells is an emerging area of cancer immunotherapy. T lymphocytes from cancer patients are engineered to express synthetic CARs, redirecting them to detect and eliminate cancer cells which express the CAR-targeted ligand [5]. These CARs consist of four components: an intracellular signalling domain, a transmembrane domain, a hinge region and an antibody-binding domain [6]. After poor outcomes in clinical trials using the first generation CAR structure, where the intracellular CD3-ζ signal was insufficient to elicit a T cell response in vivo, costimulatory signals were found to be imperative [7,8,9,10]. This modular second-generation CAR design incorporates the initiator of T cell signalling, CD3-ζ, at the intracellular domain with a costimulatory signal from CD28 or CD137 (4-1BB). Third generation CARs consist of two or more costimulatory domains. The antigen binding domain provides the CAR specificity, with the most successful CAR T cells targeting the B-cell antigen CD19, which have shown promise in the treatment of B-cell malignancies [11,12]. Traditionally, the antigen-binding domain is composed of the variable heavy and light chains of monoclonal antibodies (mAbs) which are connected by a flexible linker to form a single chain variable fragment (scFv). These scFv domains target extracellular antigens of cell-surface proteins which are expressed by cancer cells, eliminating the need for antigen presentation by major histocompatibility complex (MHC) [13] (Figure 1).

### 2.1. Development and Recent Advances CAR T Cell Therapy 

#### 2.1.1. Haematological Cancers

CAR T cell therapy utilises T cells harvested from cancer patients (apheresis) which are subsequently antibody activated and transduced with either a lentivirus or retrovirus vector. Cells expressing the synthetic CAR are expanded ex vivo before being reinfused back into the patient after lymphodepleting chemotherapy [14]. Upon antigen recognition by the CAR, targeted cell death is initiated by the activated T cell which undergoes rapid expansion and persists in the patient. The use of CAR T cells for anti-cancer therapy showed poor efficacy in the first clinical trials. Using the first-generation CAR design, these clinical trials targeting ovarian cancer, renal cell carcinoma and non-Hodgkin lymphoma, showed poor patient outcomes and suboptimal T cell expansion in vivo, eliciting poor response and in some situations extreme adverse toxicities [8,9,10].

Second-generation CAR models were then designed to incorporate a CD137 (4-1BB) costimulatory domain which has been shown to increase clonal expansion and long-term endurance of the CAR T cells, a limitation of previous clinical trials [14,15]. To date, the greatest success in using CAR T cells to target cancer cells has been achieved with targeting CD19+ B-cells in B-cell malignancies such as B-cell non-Hodgkin lymphoma (NHL), Acute Lymphoblastic Leukaemia (ALL) and Chronic Lymphocytic Leukaemia (CLL) [12]. Clinical trial results showed complete remission in 90% of B-ALL patients treated with CD19 CAR T cell, even when previous stem cell transplants were unsuccessful [14]. After great success of CD19-targeted CAR T cells in clinical trials, this therapy is now FDA-approved as routine therapy for children and adults with relapsed or refractory B-ALL or Diffuse Large B-cell Lymphoma (DLBCL) [16].

While CD19-targeted CAR T cells are successful in treating B cell malignancies, recent studies are expanding this immunotherapy to the treatment of multiple myeloma (MM). Clinical trials into targeting different myeloma antigens, such as B cell maturation antigen (BCMA), CD138, immunoglobulin light chains and CS1 glycoprotein antigen (SLAMF7) are ongoing. The most widely targeted antigen, BCMA, has shown promise in treating relapsed/refractory MM (RRMM) in phase I/II clinical trials. In the first early clinical trial using a BCMA-targeted CAR design, among the 12 patients in the trial, three patients entered partial remission, three patients had a stable disease and one patient achieved complete remission (NCT02215967). Further developments of the CAR design, still targeting BCMA but with the addition of the 4-1BB costimulatory domain, which is shown to increase T cell persistence, were then trialled and showed that clinical response was dose dependent and a 100% complete response was recorded with the higher dose of 450 × 10^6^ CAR T cells [17]. Several CAR constructs targeting BCMA are in clinical trial, with the hope that soon they will be approved for clinical therapy, improving the outcome for MM patients. 

Although CD19-targeted CAR T cell therapy shows promising results in patients with haematological malignancies, it does not come without toxicities. The best characterised toxicities have been seen in clinical trials of patients treated with CD19-targeted CAR T cells. With CD19 being expressed on all B-cells and its precursors, it is expected that B-cell aplasia occurs as a result of CD19-targeted cell killing which can persist for longer than a year post treatment depending on the follow-up time available [18]. The most prominent toxicity seen in the early CD19-directed CAR T cell therapy trials was Cytokine Release Syndrome (CRS) [14]. This is the increase in cytokine release, notably IFN-γ and TNF-α as a result of T cell activation and expansion upon interaction of T cell CARs with the target antigen. These cytokine signals activate monocytes and macrophages to release pro-inflammatory cytokines IL-1, IL-6 and IL-10. This cytokine storm leads to the clinical features of high fever, fatigue, myalgia, nausea. Severe CRS is characterised by organ dysfunction with hypertension and hypoxia [19]. More recently, a study reported CD19-targeted CAR T cell therapy induced glial cell injury in approximately 40% of patients due to CRS [20]. In addition to close monitoring of symptoms, treatment of CRS with the IL-6 blockade, Tocilizumab, has been successful in the management of CRS with only a few cases showing resistance to Tocilizumab [21].

Immune-cell associated neurotoxicity syndrome (ICANS) is the second most common toxicity and is characterised by symptoms of confusion and delirium and occasionally cerebral oedema. ICANS manifests as a result of cytokines in the brain, supported by high serum levels of IL-6 in patients showing neurotoxicity after CAR T cell treatment [21]. Similar to CRS management, monitoring symptoms closely and administering corticosteroids is recommended for the treatment of ICANS. 

##### Limitations of CAR T Cell Therapy for Haematological Cancers

One limitation of using CD19-directed CAR T cells in the treatment of B-cell malignancies is the relapse that occurs of CD19^-^ B-cells which escape the first treatment. As more patients are treated with CD19 CAR T cells and more follow-up data becomes available, it is understood that 39% of patients relapse with a CD19^-^ disease [22]. Fousek et al. has studied the use of bi-directional and tri-directional CARs which as well as CD19, target CD20 and CD22, two other B-lineage markers [23]. After promising in vitro studies which showed success in targeting CD19− escape B-ALL cells, in vivo mouse models were used to investigate the efficacy of CD19/20/22 CAR T cell therapy on targeting CD19+ and CD19− escape malignancies. Treatments of CD19/20/22 CAR T cells showed significant anti-tumour response in both mouse models with CD19+ B-ALL as well as the CD19− escape B-ALL which CD19 CAR T cells was ineffective at treating alone [24]. This method presents an exciting branch of CAR T cell therapy for haematological B cell malignancies which do not express CD19. 

Other approaches have targeted tyrosine-protein kinase transmembrane receptor 1 (ROR1), a glycosylated type 1 membrane protein expressed on the surface of malignant cells, including ALL and CLL cells, but not on normal B-cells [25,26]. Targeting ROR1 may reduce ‘on-target, off-tumour’ toxicities compared to CARs targeting CD19/20/22, however, it has been found that ROR1 is expressed on pancreatic and lung tissue suggesting potential for other toxicities [27]. Phase I clinical trials have begun using ROR1-CAR T cells although results are yet to be obtained (NCT02194374). 

The use of CAR T-cells as treatment for haematological cancers is a costly and complex process, as T-cells are derived from individual patients and engineered to ensure HLA matches. Liu et al., 2020, has investigated the use of natural killer (NK) cells which omit the need for this autologous method as HLA matching is not necessary. This phase I and phase II study of 11 patients with CD19- positive haematological cancers utilised a retroviral vector that expressed anti-CD19 CAR with IL-15 and Caspase 9 to engineered NK cells. Patients were treated with lymphodepleting chemotherapy with Fludarabine and cyclophosphamide, followed by single infusions of CAR-NKs at one of three increasing concentrations. Early data shows that CAR-NK cells had better anti-tumour activity and persistence than non-transduced NK cells, with seven of 11 patients (64%) showing a complete response after a mean follow-up of 13.8 months. NK cells persisted in the patients for at least 12 months, potentially a result of the inclusion of IL-15 in the retroviral vector. This study presented a new method of treating haematological cancers without leading to the toxicities associated with CAR T-cell therapy, such as CRS and neurological ICANS [28].

A lack of CAR T cell persistence is an issue in both haematological cancers and solid tumours. The persistence of CAR T-cells within the patient is influenced by several factors including starting T-cell population, T cell exhaustion, host immunogenicity but is also influenced by the CAR design, a target for aiming to improve CAR T cell persistence. Incorporating structures into the CAR design, such as 4-1BB, has shown to increase T-cell proliferation and persistence. Human CAR constructs are also preferred in updated CAR designs which reduce immune-mediated rejection of the CAR, allowing the T-cell to persist [29]. While CAR-NK cells can overcome the issue of T-cell persistence patients, adapting the CAR construct can also increase the efficacy of CAR T-cell treatment for both haematological and solid cancers. 

#### 2.1.2. Solid Tumours—Challenges and Developments

While CAR T cell therapy has been greatly successful in the treatment of B-cell malignancies, barriers arise when translating this therapy to solid tumours, reviewed elsewhere [30]. There are three main factors hindering the use of CAR T cells for the treatment of solid cancers; firstly, the desmoplastic nature of the solid TME creates a physical barrier for T cells to enter [31]. This creates trafficking issues hindering the activity of the CAR T cells targeting the solid tumour. Secondly, the antigens expressed on tumour cells are heterogeneous and are expressed broadly but in low levels across normal tissue, limiting the number of potential targets which do not elicit severe toxicity. Thirdly, the TME of advanced cancers is often immunosuppressive and leads to T cell exhaustion and anergy [32]. It is these barriers which prevent translating the use of CAR T cell therapy to solid tumours but have focused research in understanding ways to overcome these barriers. 

Due to the physical barrier and suppressive nature of the TME, the mode of application of the CAR T cells is important to ensure optimal penetrance into the tumour tissue and thus increased anti-tumour activity. With this in mind, studies are focusing on three areas: the CAR design, the host response and the T cell subset, aiming to increase T cell trafficking, penetrance and persistence. To overcome the physical barrier of the TME and enhance T cell trafficking into the TME, one method has taken CAR T cells and engineered them to express IL-8 receptors CXCR1 or CXCR2 [33]. Tumours can overexpress IL-8 naturally but ionising radiation also increases the production of IL-8, and cells expressing the receptor for IL-8 follow a chemotaxis gradient towards the IL-8 secreting cells. In this study, CD70-directed CAR T cells were engineered to express IL-8 receptors which aims to enhance intratumour chemotaxis of the T cells when IL-8 is present [33]. These 8R70CAR T cells showed significant anti-tumour responses in glioblastoma, ovarian and pancreatic cancer in vivo mice models, leading to complete regression of larger and late-stage tumours as well as prolonged long-term protection [33]. Additionally, these 8R70CAR T cells have shown further successes in anti-tumour properties by reducing the level of the pro-cancer chemokine in the process of T cell trafficking. The success of this strategy is progressing onto human phase I clinical trials. Similar efforts have also been studied in upregulating the expression of IL-23 receptors in CAR T cells [34]. In another recent study, switchable CAR T cells have been used to target receptors expressed across several cancers but also across normal tissue. These CAR T cells are directed towards the HER2 receptor but are engineered to target an antibody based switch which acts as a bridge between the CAR T cell and the HER2 on the target cell [35,36]. Preclinical trials of switchable CAR treatment have been successful in mouse xenograft models of breast, ovarian and pancreatic cancers where a significant reduction in tumour volumes was observed [37]. Survival of the engineered CAR T cells within the tumour microenvironment is essential for an anti-tumour response. Adachi et al. (2018), showed how engineering the CAR design to express IL-7 and CCL19, both essential for the maintenance of T cell zones in lymphoid organs, increases the infiltration of dendritic cells and T cells into the tumour tissue. This led to an increased regression of solid tumours without recurrence, where the anti-tumour effect of these 7 × 19 CAR T cells proved to be more efficient than conventional CAR T cells [38].

One approach to overcome the heterogeneity of solid tumours is to target antigens present across malignant and normal cells, but which are either mutated or differentially expressed in the tumour setting. An example of this is targeting MUC1, a highly glycosylated transmembrane mucin protein. In a tumour setting, aberrant glycosylation of MUC1, seen in 90% of breast cancers, exposes epitopes that would not normally be accessible for recognition in normal tissue [39]. Developing MUC1-directed CAR T cells, Zhou et al. investigated the efficacy of this treatment on mice models orthotopically injected with HCC70 breast cancer cells [39]. The results of this in vivo study showed that the MUC1-directed CAR T cells resulted in significantly reduced tumour size which persisted throughout the study length. Long term efficacy was also recorded after treatment with MUC1-directed CAR T cells [39]. With MUC1 being aberrantly expressed in a variety of cancers, such as Pancreatic Ductal Adenocarcinoma, its translation to other cancer is being explored.

Targeting two or more antigens is also a promising method for the treatment of solid tumours. The use of bispecific CAR T cells has been investigated in glioblastoma, targeting HER2 and IL-13Rα2 in a combinational design [40]. The co-expression of HER2 and IL-13Rα2 means that the tumour cell must express both for the T cell to become activated to kill the cancer cell and promising results have been found in vitro and in vivo studies.

The TME consists of myeloid derived suppressor cells (MDSC), tumour associated macrophages (TAM) and regulatory T cells (T_regs_) which function to supress the action of T cells in eliminating tumour cells [41]. This is an issue when it comes to using T cells to target cancer cells and so using armoured CAR T cells has proven effective in overcoming the immunosuppressive nature of the TME [42]. These Muc16-directed T cells are engineered to secrete IL-12 upon antigen stimulation. This aims to enhance the cytotoxic capability of CD8+ cells as well as reduce antigen escape in CAR T cell treatment by recruiting macrophages to the TME. This method has successfully gone to phase I clinical trial (NCT02498912) after reporting that treatment lead to complete and persistent response in mouse models of ovarian peritoneal carcinomas (Table 1).

These recent advances in CAR T cell therapy directed towards solid tumours show the committed length research is taking to overcome these translational barriers, and it is the modular design of synthetic CARs which allow for adaptations to be tested. While there are several areas of immunotherapies which show increasing success in the treatment of solid tumours, it may be the combination of these immunotherapies which show the most promising results. 

#### 2.1.3. Combination Treatment with CAR- T Cell Therapy

T cell exhaustion after reinfusion of the CAR T cells into the patient still presents as the biggest limitation facing CAR T cell therapy. Methods to overcome this have investigated the use of immune checkpoint inhibitors in combination with the CAR T cell therapy in ways of either co-administration or engineering the CAR T cells to secrete the monoclonal checkpoint blockade antibody. The first method of co-infusion of CAR T cells with checkpoint blockade Abs is being investigated in glioblastoma patients, where patients will be treated with a second generation epidermal growth factor receptor variant III (EGFRvIII)-directed CAR T cells with the co-administration of Pembrolizumab, a PD-1 monoclonal antibody checkpoint inhibitor (NCT03726515). While results of this phase I clinical trial are yet to be obtained, previous phase I clinical trials have shown success in the use of EGFRvIII-directed CAR T cell therapy alone and preclinical studies combining these therapies have been successful in vivo mouse models, showing increased tumour killing activity with specific immune checkpoint blockades [43].

A second phase I/II clinical trial is investigating combining CAR T cell therapy with immune checkpoint blockade by engineering EGFRvIII-directed CAR T cells to express anti- CTLA-4 and PD-1 Abs (NCT03182816). Previous studies have investigated engineering CAR T cells in this way, which was successful in improving the persistence and activity of T cells in its cancer killing capacity [44].

The combination of CAR T cell therapy with more conventional chemotherapies and radiotherapy has been investigated in several clinical trials. Alone, CAR T cell therapy and chemotherapy are not sufficient to eliminate solid tumours, without any recurrence but together, it has been shown that chemotherapy agents are successful in improving the efficacy of CAR T cells. Certain chemotherapies, such as cyclophosphamide, gemcitabine and doxorubicin, can inhibit the suppressive immune cells which prevent CAR T cell function. Inhibition of T_reg_ cells enhanced the efficacy of anti-tumour immunotherapy as the CAR T cells are able to persist in the TME for prolonged times [45]. The addition of radiotherapy has also been shown to amplify the efficacy of CAR T cell therapy, enhancing T cell trafficking and penetrance into the tumour microenvironment [46]. A recent phase I clinical trial of patients with either glioblastoma or gliosarcoma has studied the use of EGFRvIII CAR T cells prior to standard radiotherapy (NCT02664363), with the aim that the combination increases complete response compared to CAR T cell or radiotherapy alone [47].

These early stage clinical trials show the promise that combining CAR T cell therapy with other immunotherapies, chemotherapies or radiotherapies has on the treatment of solid tumours, which previously faced several barriers.

## 3. Immune Checkpoint Inhibitors

Immune checkpoint inhibitors are therapeutic Abs that work by inhibiting the interaction between negative immunologic regulators and their binding partners. Immune checkpoints refer to the inhibitory pathways crucial for the maintenance of self-tolerance and of the immune responses [48]. In normal physiological conditions, their main role is to act as a natural break to the normal host immune response, acting as a negative regulator of the amplitude and quality of T cell activation to prevent an aggressive immune reaction and minimize damage to healthy cells in the body [49]. 

T cells, to be fully activated, require multiple steps which involve clonal selection of antigen-specific cells, activation and proliferation in lymphoid tissues to then execute their effector functions at the target tissue [50]. Each of these steps is regulated by immune checkpoint proteins [51], of which, two candidate proteins that have been more extensively and actively studied are the programmed cell death protein 1 (PD-1) and cytotoxic T-lymphocyte-associated antigen 4 (CTLA-4). These are inhibitory receptors induced on the surface of T cells after the initial steps of T-cell activation in the lymph nodes (CTLA-4) or reduce T-cell activity in the target tissue (PD-1) [52]. 

However, it has been demonstrated that tumour cells or non-transformed cells in the TME also overexpress these inhibitory immune checkpoint proteins which prevent T cells from destroying the target cancer cells [53]. For this reason, these immune checkpoints can be blocked using specific and selective antibodies (Abs) that target either the receptor or their ligands to increase antitumor T-cell activity [48]. Interestingly, immune checkpoint inhibitors are not restricted to T cells. In fact, NK cells are a newly emerging target. NK cells are innate lymphocytes specialised in early defence against transformed cells [54]. Their function is regulated by activatory and inhibitory surface receptors (checkpoint molecules). Alterations in the expression of these receptors can result in impaired cytotoxicity against tumour cells [55]. Thus, it is now of interest to target NK-cell receptors with monoclonal antibodies for cancer immunotherapy. However, in this review, we discuss checkpoint inhibitors in modulating T cell response against cancer.

### 3.1. Development of Immune Checkpoint Inhibitors

#### 3.1.1. Cytotoxic T-Lymphocyte-Associated Antigen 4—CTLA-4 

**Figure 2:**
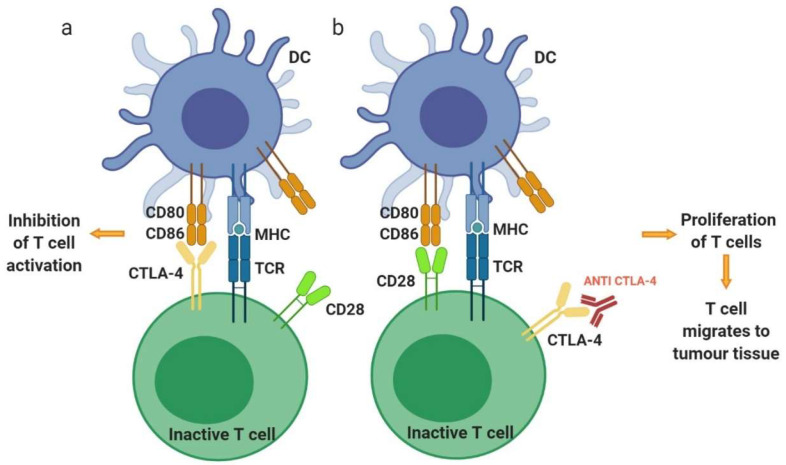
Schematic representation of CTLA-4 and anti-CTLA-4 mechanisms of action on T cell activation. (**a**) Following the binding of MHC-presented immunogenic peptide antigen to the TCR, the co-inhibitory cell surface receptor CTLA-4 will bind to its ligands CD80 and CD86 found on antigen presenting cells, blocking the co-stimulatory signal (brought by CD28) thus preventing continued T cell activation. (**b**) By blocking CTLA-4–CD80 or CTLA-4–CD86 interaction with anti-CTLA-4 Abs, T cells proliferation will be activated and will migrate towards secondary lymphoid organs.

The first checkpoint antibody approved by the FDA was ipilimumab (CTLA-4 inhibitor) for the treatment of melanoma. CTLA-4 (also known as CD152) is a member of the immunoglobulin superfamily, B7/CD28 [56]. It is constitutively expressed on T_regs_ but can also be upregulated by other T cell, especially CD4+ T cells, following activation [48,52]. Their main role is to counteract the activity of the co-stimulatory receptor CD28. Both CTLA-4 and CD28 share the same ligands, CD80 and CD86. CD28 is constitutively expressed on the T cells and interaction between CD28 and CD80/86 causes the T cell receptor (TCR) signals to amplify and leads to T cell activation [57] (Figure 2). However, CTLA-4 has a much higher overall affinity for CD80/86 and drastically reduces the activation of T cells [58]. Research has shown that CTLA-4 is also expressed on the surface of tumour cells [59] and inhibit T cell activation and prevent anti-tumour immunity. Partial blocking of CTLA-4 with Abs resulted in regression of tumour in mice displaying partially immunogenic tumours [60]. These findings in preclinical studies led to the production of fully humanised CTLA-4 Abs, ipilimumab and tremelimumab. Clinical testing was initially carried out on patients that were not responding to conventional treatments and with cancers in advanced stages [61]. The outcome was that both ipilimumab and tremelimumab produced a clinical response in 10% of the patients with melanoma [62]. Despite both humanised Abs reached phase III clinical trials, ipilimumab (Yervoy) was the only drug approved for the treatment of advanced melanoma by the FDA in 2010. Ipilimumab has since been extensively investigated as a therapeutic agent in several clinical trials; for example, Yervoy showed promising results in a phase I/Ib multicentre, investigator-initiated study where the efficacy of ipilimumab in patients with relapsed hematologic cancer after allogeneic HSCT was investigated [63]. Its approval by the FDA came after the results of a randomized three-arm clinical trial (NCT00094653) of patients with advanced melanoma where patients receiving ipilimumab showed a median overall 10-month survival benefit [64]. However, survival analysis at a later point revealed a reduced survival rate to 23.5% after two years of treatment compared to 45.6% after year 1 of treatment [64]. This suggests that a substantial proportion of initial-responders relapse probably with lethal, drug-resistant disease months or years later [65], which emphasises the importance of targeting other factors such as PD-1/PD-L1, and introducing more efficacious therapy and/or a combination approach. 

#### 3.1.2. Programmed Cell Death Protein 1—PD-1

**Figure 3:**
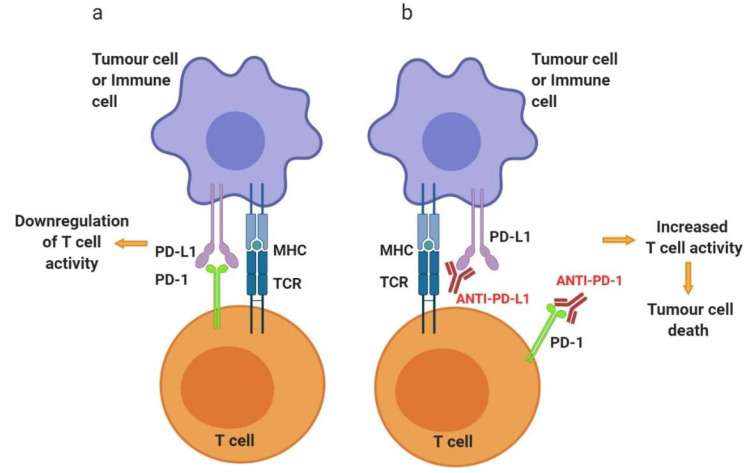
Schematic representation of PD-1 and anti-PD-1/PD-L1 mechanisms of action on T cell activity. Activated T cells at secondary lymphoid organs/tumour tissue (**a**) will upregulate the expression of co-inhibitory cell surface receptor PD-1. Binding of PD-1 to its ligands, PD-L1 or PD-L2, found on the surface of several immune cells as well as tumour cells, will inhibit signalling downstream of the TCR, thus downregulating T cell activity. (**b**) Targeting PD-1 or PD-L1 with antibody therapeutics can reinvigorate exhausted T cells at the tumour site, increase the activity, consequently allowing T cell-mediated tumour cell killing.

PD-1, also known as CD279, is a type I transmembrane protein receptor predominantly expressed on T cells, B cells, natural killer (NK) T cells, activated monocytes, and dendritic cells (DCs) [66]. Their main role is to regulate T cell mediated immune response in peripheral tissues, mounting a strong resistance mechanism within the TME [67]. PD-1 has two ligands: PD-1 ligand 1 (PD-L1) and PD-1 ligand 2 (PD-L2) (Figure 3), both members of the B7 family and they bind to a co-stimulatory receptor on T cells [68]. In physiology, activated T cells demonstrate an upregulation of PD-1 which lasts until these cells reach the target tissue. Here, PD-1 ligands—PD-L1 and PD-L2—expression is induced leading to the downregulation of T cell activity, thus reducing damages to the tissues possibly caused by a strong immune response [48]. It is not surprising that, due to its important role in the prevention of autoimmunity, the PD-1 signalling pathway can be used by tumour cells to evade the antitumor immune response [69]. It has been demonstrated that PD-1 is highly expressed on tumour-infiltrating lymphocytes (TILs) and its ligands, particularly PD-L1, are constitutively expressed on tumour cells of various types e.g., melanoma and lung cancer cells [70]. In addition, PD-L1 is often expressed on myeloid cells within the TME which also contributes to the inhibition of antitumor immunity [71]. Studies showed that if either tumour cells or TILs expressed PD-L1, the overall survival is decreased compared to PD-L1 negative tumours [48]. 

Based on this finding and the relationship between PD-1 and PD-L1, Abs were developed to specifically inhibit these molecules to enhance the host immune response against cancerous cells. For instance, animal studies demonstrated that by blocking either PD-1 or its ligands, the antitumor immune response was amplified [72]. Thus, several PD-1 and PD-L1 inhibitors have been or are being tested for use in several different cancers such as non-small cell lung cancer (NSCLC), head and neck squamous cell carcinoma (HNSCC) and Hodgkin lymphoma. Examples of drugs that have been approved by the FDA targeting PD-1 are shown in Table 2.

Many clinical trials are currently ongoing in a variety of cancer types. For instance, phase III trials for patients with cholangiocarcinoma have been initiated in China (NCT03101488) to investigate Envafolimab, a first-in-class nanobody that binds with high affinity and specificity to PD-L1, blocking interaction with PD-1, and resulting in T cell-mediated immune response to neoplasms.

Although in clinics, anti-PD-1/PD-L1 therapies pose a number of adverse effects and toxicities such a cardiotoxicity, myocarditis, hepatitis, diarrhoea, endocrine dysfunction, etc., reviewed elsewhere [79,80]. Anti-PD-1 therapies have shown to trigger TNF production, which potently impairs CD8+ TIL response. There are now guidelines available on the management of adverse events in anti-PD-1/PD-L1 therapies [81,82,83]. Additionally, anti-PD-1/PD-L1 therapies can develop resistance. For instance, in a follow-up study involving 205 melanoma patients treated with pembrolizumab, 26% of patients developed resistance. A similar outcome was observed in melanoma patients where 15 out of 42 patients developed a 35% resistance rate [84].

Another factor that remains unknown is the optimal duration of treatment with checkpoint inhibitors [85]. For instance, longer treatment duration is generally associated with higher incidences of immune-related selected toxicities [85]. Although most clinical trials with checkpoint inhibitors have been carried out over a two-year duration, investigations are underway to identify shorter optimal duration with better clinical outcome, least amount of toxicities and reduced burden of financial toxicity [85]. This is even more relevant to the current pandemic, COVID-19, due to its added risk to patients life and the over-representation of cancer patients (e.g., lung cancer patients representing up to 28%) among those affected by the impact of SARS-COV-2 [86].

These challenges, along with the limited benefits of these checkpoint inhibitors, meant that better treatment plans were required in place and hence, a combination of anti-PD-1/PD-L1 and anti-CTLA-4 therapies were tested in both pre-clinical and clinical settings.

#### 3.1.3. Combination Therapy for CTLA-4 and PD-1 

Although overall survival of patients treated with anti-CTLA-4 increased, only a limited number of patients benefit from this in the long term. Thus, efforts are being directed towards finding biomarkers that can predict responses to anti-CTLA-4 [53], as well as evaluating the efficacy of the CTLA-4 inhibitor (ipilimumab) in combination with other immune checkpoint inhibitors. For instance, in a follow up report of the CheckMate067 (phase III trial), a significant improvement in overall survival and objective response rate was observed in advanced melanoma patients treated with nivolumab (PD-1 antibody) plus ipilimumab (CTLA-4 antibody) compare to each monotherapy [87]. Twenty-one percent of the patients who received the combination therapy achieved complete remission, whilst 37% of the patients had 30% reduction in lesions and achieved partial remission. Unfortunately, 59% of patients in this group reported severe adverse events [87]. Thus, although a combination of these therapies show promise, they also come with enhanced toxicities, and associated costs. Interestingly, in a promising pre-clinical study it was shown that using anti-TNF antibody or TNF-receptor inhibitor with this combination therapy can significantly reduce increased double-checkpoint blockade-induced colitis and increase infiltration of tumour-specific CD8+ T cells in to the TME [88,89]. A phase I trial (NCT03293784) in metastatic melanoma patients is in progress.

It has been shown that some patients respond better than others to combination treatments because of a number of possible factors such as the composition of their gut microbiota [90], mutational signatures or aneuploidy in the tumour [91,92]. For instance, when researcher addressed the role of the gut microbiota of patients with metastatic melanoma was investigated, they observed that mice that received faecal microbial transplantation of faeces harvested from individuals with abundance of immunogenic *Bacteroides spp.* in the gut (39%), showed a greater reduction in tumour size after ipilimumab treatment. This indicated that a certain composition of the gut microbiota is connected to a better anticancer response [90]. Additionally, recent investigations in the human leukocyte antigen class I (HLA-I) of cancer patients—advanced melanoma and NSCLC—indicated that reduced survival following checkpoint blockade therapy is associated with homozygosity at HLA loci, suggesting polymorphisms in the HLA genes may underpin responsiveness to immune checkpoint inhibitors. Moreover, it was observed that the presence of the HLA-B62 supertype (including HLA-B*15:01) is correlated with a poor survival as they impair the ability of CD8+ TCR to recognise neoantigens [93]. Thus, these results need to be confirmed and used to stratify which patients should receive immune checkpoint therapies.

Currently, several clinical trials involving both anti-PD-1 and anti-CTLA-4 have reached phase III of the trials. One of these is the Checkmate 649 trial (NCT02872116) for gastric cancer/gastroesophageal junction cancer. This clinical trial was designed based on a previous multicentre, open-label, phase I/II trial (CheckMate 032; NCT01928394) with nivolumab and nivolumab/ipilimumab in the second-line setting. On March 2020, following the success of a multicentre, multiple cohort, open-label trial (NCT01658878), the FDA approved the use of ipilimumab in combination with nivolumab (OPDIVO, a anti PD-L1 drug) for patients with hepatocellular carcinoma (HCC) who have been previously treated with sorafenib [94]. This is an interesting bispecific targeting strategy which should enhance anti-tumour response by the host immunity.

### 3.2. Beyond PD-1 and CTLA-4

Despite the success of the previously mentioned immune checkpoint therapies, only a small percentage of patients (10–30%) show durable responses [95]. In fact, many patients develop de novo or adaptive resistance, as well as severe immune-related adverse events (irAEs). For this reason, research has recently focused on finding novel immune checkpoint targets with the intent of using them either in monotherapy or in combination with other immune checkpoints inhibitors. Some promising therapeutic targets that are currently being characterised and under clinical trials are the lymphocyte activation gene-3 (LAG-3) [96], the T cell immunoglobulin and mucin-domain containing-3 (TIM-3) [97] and the T cell immunoglobulin and ITIM domain (TIGIT) [98]. 

#### 3.2.1. Lymphocyte Activation Gene-3 – LAG-3

LAG-3 (CD223) was first discovered in the early 1990s by Triebel et al. [99]. It is expressed on several cell types including CD4+ and CD8+ T cells [99], T_regs_ [100] and a subpopulation NK cells [101]. Evidence has shown that LAG-3 signalling is responsible for negatively regulating the activation and proliferation of T helper 1 (Th1) cells, and cytokine secretion [102]. Several ligands that interact with LAG-3 have been identified, such as MHC-II, galectin-3, LSECtin, a-synuclein, and fibrinogen-like protein 1 (FGL1) [103]. It has been shown that a constant stimulation of antigens in cancer or during an infection results in LAG-3 being chronically expressed, leading to T cell exhaustion [95]. Thus, targeting LAG-3 can potentially facilitate T cell reinvigoration. Based on promising experimental results, the first clinical trials concentrated on developing an antibody sLAG-3-Ig, IMP321 (Eftilagimod alpha), which showed only modest clinical responses in patients with metastatic renal cell carcinoma (mRCC) [104]. However, the first mAb directed against LAG-3 to be commercially available is relatlimab for the treatment of melanoma [105]. The first trial in which relatlimab was involved was to evaluate its efficacy as monotherapy or in combination with the anti-PD-1 nivolumab (NCT01968109) [106]. This showed an overall response rate of 11.5%, and even higher in patients with higher LAG-3 expression (≥1%) [107]. Currently, there are more than 18 registered clinical trials working on relatlimab, some in phase I or II, but none completed. 

##### 3.2.2. T Cell Immunoglobulin and Mucin-Domain Containing-3—TIM-3

TIM-3 (HAVCR2) is a member of the TIM family and has been known to express mainly on CD4+ Th1 and CD8+ t cytotoxic 1 cells as well as on B cells, T_regs_, NK cells, DCs, macrophages and monocytes [108]. TIM-3 interacts with numerous ligands including tumour-secreted galectin-9, high-mobility group protein B1 (HMGB1), carcinoembryonic antigen cell adhesion molecule 1 (CEACAM-1, expressed on tumour cells), and phosphatidyl serine (PtdSer) [109]. The upregulation of TIM-3 is linked with poor prognosis in solid tumours and some preclinical trials have shown that the blockade of TIM-3 leads to the reduction of tumour growth [103]. Considering the promising results, several clinical trials have been initiated to evaluate the efficacy of TIM-3 antagonistic mAbs. One of these is a multicentre, open-label study (NCT02817633) intended for studying a novel IgG4 anti-TIM-3 mAb, TSR-022 (Cobolimab), as a monotherapy or in combination with anti-PD-1 mAb in patients with advanced solid tumour which showed clinical benefits in the combination cohort [110] with a 15% objective response rate and 40% stable disease [111].

##### 3.2.3. T Cell Immunoglobulin and ITIM Domain—TIGIT

TIGIT, also known as WUCAM and Vstm3, is a member of the CD28 family [112]. It was first identified as an immune checkpoint receptor with its expression limited to NK cells and T cells subsets (T_regs_ and memory T cells) [98]. TIGIT binds two ligands: CD155 and CD112 [113] which are shared with other counterparts CD266 and/or CD96. Depending on which receptor binds the ligands, either a positive co-stimulatory signal (CD226) [98] or inhibitory signals (TIGIT) are produced [114]. Based on preclinical trial results, several pharmaceutical companies started to develop anti-TIGIT drugs and are currently in early phase clinical trials. One of these is Etigilimab (OMP-313 M32), a humanized mAb created to prevent the interaction between TIGIT and CD155. This is an open-label research (NCT03119428) to study the safety and efficacy of the anti-TIGIT both as monotherapy and in combination with an anti-PD-1 mAb in patients with advanced malignancies [115].

Despite the numerous clinical trials researching these new targets, no drug is available in clinic at this date.

## 4. Dendritic Cell Vaccines

**Figure 4:**
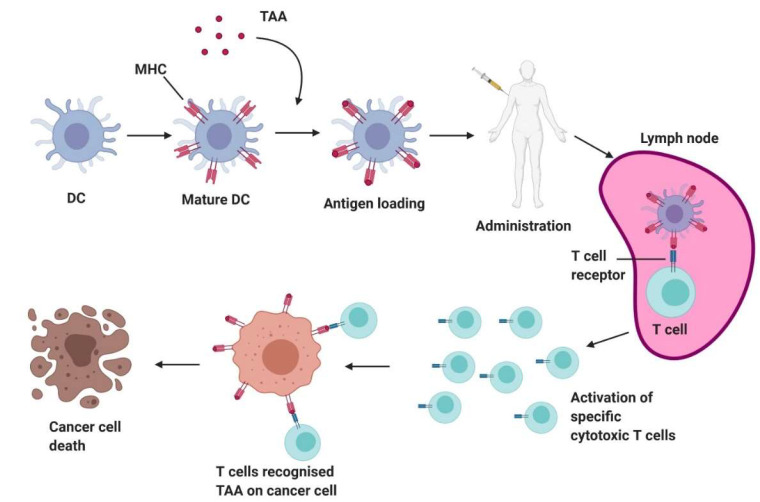
Action of DC vaccine in the body. Dendritic cells are matured and loaded ex vivo with tumour associated antigens (TAA). Following administration of the vaccine, antigen-specific T cells are activated and circulate round the body searching for cancer cells expressing their respective antigen. After detecting a cancer cell, T cells dock and exert their cytotoxic activity.

Dendritic cells (DCs) are antigen-presentation cells that ‘present’ peptides to T-cells leading to their activation. DCs can therefore be cultured ex vivo with specific tumour antigens and reinfused into the patient to generate potent cancer vaccines. Unlike other therapies, DC-based vaccines function by boosting the patient’s own immune response against their own tumour and pose a low risk of toxicity [116]. DC vaccines have been tested in phase I, II and III clinical trials for a variety of cancers, including melanoma [117], AML [118], myeloma [119], HNSCC [120] and ovarian cancer [121]. Naturally-circulating DCs or monocytes are isolated from the patient’s blood via leukapheresis and cultured in vitro with a specific cocktail of cytokines, depending on the cancer and type of T cells needed to be activated, to induce differentiation into mature DCs. Pulsing the DCs with antigen peptide, protein, mRNA or tumour cells/lysate prime the cells for the specific antigen(s) expressed by the tumour. The vaccine is injected into the patient, resulting in the activation of antigen-specific T cells [122] (Figure 4).

### 4.1. Development and Recent Advances of DC Vaccines

Based on the native capacity of potent antigen presentation and T cell activation, and its ease of development in vitro conditions, DCs were tested as cancer vaccine around mid-1990s. However, it was in 2010, when the FDA approved the first DC vaccine, Sipuleucel-T, a treatment for asymptomatic castration resistant prostate cancer [123]. This study highlighted that DC vaccines are safe and well tolerated in cancer treatment; however, limited clinical benefit was seen when treating patients with advanced prostate cancer.

This limited efficacy was thought to be due to advanced/metastatic tumours deploying a number of immunosuppressive strategies that prevent the maturation and activation of DCs. High expression levels of immunosuppressive cytokines (VEGF, TFGβ) are exhibited by advanced tumours [124,125], which prevent DC differentiation and maturation, creating an immunosuppressive microenvironment in which tumour cells thrive. Increased expression of suppressive alarmins, e.g., matrix metalloproteinase-2 (MMP-2) is also detected in cancer cells [126]. These digest the extracellular matrix, aiding tumour invasion and inhibit secretion of IL-12, preventing Th1 T cell differentiation and NK cell activation and, hence, a high level of MMP2 is associated with a poor prognosis. Therefore, recent research into DC vaccines has been aimed at improving DC activation and promoting of T cell function, as well as the use of adjuvant treatments alongside DC vaccines to counteract the effect of an immunosuppressive TME [127].

**Figure 5:**
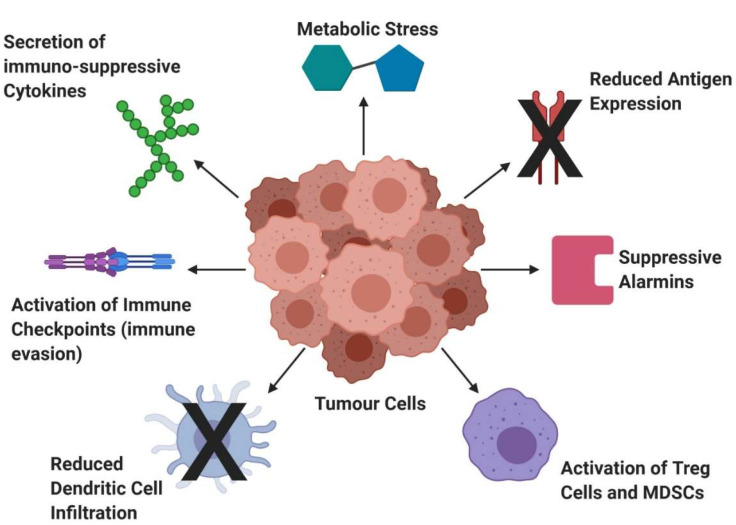
Dendritic cell dysfunction in cancer. Tumour cells have the ability to alter the microenvironment to impair the function of dendritic cells (DCs), suppressing an anti-tumour immune response. Metabolic stress: Tumour cell can decrease the availability of nutrients and oxygen in the tumour microenvironment (TME), altering DCs metabolism and impairing their function. Reduced antigen expression: Tumour cells have the ability to alter/hide their antigens to avoid detection by the immune system. Suppressive alarmins: Expression of alarmins, e.g., MMP-2 have been found to create an immunosuppressive environment by inhibiting the secretion of IL-12 by DCs thus preventing Th1 T cell differentiation and NK cell activation. Activation of T-ref cells and myeloid-derived suppressor cells (MDSCs): Tumour cells are able to directly induce the activation of T_reg_ cells and MDSCs which function to suppress the immune system by inhibiting T cell production. Reduced Dendritic Cell Infiltration: Tumour cells can reduce the expression of DC chemoattractants e.g., (CC-chemokine ligand 4). Activation of Immune Checkpoints: Tumour cells hijack the immune checkpoints to prevent detection. Overexpression of CTLA-4 and PD-1 ligands reduce the amplitude to T cell activation. Secretion of Immuno-suppressive cytokines: certain cytokines (IL-6 and IL-10) prevent the maturation and activation of DCs.

The most recent clinical trials surrounding DC vaccines focus on improving the ex vivo steps required to make the vaccine in order to increase efficiency [128]. These improvements aim to diminish the effects of the TME (Figure 5). Strategies include refining activation and mobilizations, maturation, dose and administration of DCs as well as creating vaccines using different subtypes of DCs.

Choosing the correct DC when creating a vaccine is vital for its success as each subtype has different capacities for antigen presentation, cytokine secretion and migration, and thus can activate different types and numbers of T cells. DCs are heterogeneous and consist of four major subsets: monocyte-derived DCs (MoDCs), plasmacytoid (pDCs; major producers of anti-viral IFN-I) and conventional DCs (cDCs; lymphoid-tissue resident), which can be further split into type 1 (cDC1s) and type 2 (cDC2s) [129]. The majority of DC vaccines are generated using MoDCs; a group of DCs that arises in response to inflammation [130]. MoDCs display features such as the ability to activate both CD8+ and CD4+ T cells, to produce co-stimulatory cytokines and migrate [127,131]. Phase II clinical trials utilising MoDCs-based vaccines have recently begun for bladder cancer (NCT04184232), endometrial cancer (NCT04212377) and advanced melanoma (NCT03803397). A recent study, focusing on treating head and neck cancer using a dendritic cell vaccinate pulsed with Wilms’ Tumour-1 peptide in combination with chemotherapy demonstrated that MoDC vaccine has the ability to enhance peptide specific immunity [120]. The vaccine was administered with OK-432, a dead/weaken form of *Streptococcus pyogenesis* used to increase DC activation. No severe side effects above grade 2 adverse events were seen in the 11 patients participating in the study; the most common side effects being a low-grade fever and mild redness around the injection site. This showed that MoDC vaccines are a safe and feasible option for advanced HNSCC when used in combination with conventional chemotherapy [120].

Despite the promising outcome of the aforementioned study, MoDCs have been reported to have decreased migration and MHC molecule expression compared to other DC subtypes, possibly due to their long ex vivo culturing periods [132]. It has been suggested that using naturally occurring DCs (pDCs and cDCs) to generate vaccines may result in a more effective treatment as they require a shorter in vitro maturation phase, preserving their innate migration and T cell activation ability [133]. pDCs are specialised in the production of type I interferons which function to activate innate immune cells e.g., NK cells and macrophages [134]. In contrast, cDCs are specialised for cross-presentation on MHC I molecules, resulting in the activation of CD8+ T cells [135,136]. Cross-presentation has proven to be vital for tumour rejection [137]. As naturally occurring DCs account for a small proportion of peripheral blood cells (<1%), the process of extracting them from the blood is a labour-intensive process, which is prone to failure [133]. Therefore, there needs to be more research into developing a standardised protocol for vaccine generation, as current treatment using naturally-occurring DCs is limited to highly specialized institutions.

On Clinicaltrials.org there are currently seven clinical trials being conducted treating patients with melanoma, prostate or any solid tumour with pDC vaccines. One trial focuses on treating melanoma stage IV patients using pDC (NCT01690377), whereas three trials focus on treating melanoma stage IV (NCT03747744, NCT01690377) or solid tumours with CD1c+ myeloid DCs (NCT03707808), the rest are testing the effects of a combination of vaccines on melanoma stage III (NCT02993315, NCT02574377) and prostate cancer (NCT02692976). Of these seven, three clinical trials have been completed. A phase I clinical trial involving 15 patients with metastatic melanoma who received intra-nodal injections of pDCs vaccines demonstrated that vaccination using pDC is feasible and results in minimal toxicity [117]. Several of the patients showed increased proliferation of CD8+ and CD4+ T cells following vaccine administration and a temporal increase of IFN secretion showing the vaccine induces favourable immune responses [117]. Similar results were seen when treating metastatic melanoma patients with primary myeloid DCs vaccines [138]. In this case, administration of the vaccine resulted in an anti-tumour response improved progression free survival.

Given that each subset of DC has different function and crosstalk abilities it has been implied that the most efficient vaccine would include multiple subsets, however there has yet to be any data generated/clinical trials completed to support this theory [139]. Additionally, one of the disadvantages of using autologous DCs in vaccines is that it takes a long time to extract them from a patient’s blood, which can be reduced by utilising allogenic DCs [140]. There is currently a clinical trial testing a pDC vaccine generated using pDCs from an allogenic cell line in melanoma patients (NCT01863108).

### 4.2. Main Challenges with DC Vaccine

Despite the promises and the safety profile, developing and administering DC vaccines can be challenging. Some of these challenges are discussed below:

#### 4.2.1. Antigen Selection and Loading

Tumour associated antigens (TAA) are critical parts of DC vaccines as they are expressed on the surface of mature DCs and are recognised by specific cytotoxic T cells. Self-antigens are found on the surface of all cells but are typically overexpressed in cancerous cells [121]. Due to their presence in normal and germline tissues, TAA prevent the generation of a strong immune response due to central tolerance. This may contribute to reduced efficiency of self-antigen DC vaccines [141].

The issue of central tolerance can be solved by incorporating neo-antigens into the production of DC vaccines. Neo-antigens contain somatic mutations specific to an individual’s cancer cells, generated by the genetic instability of the tumour [142]. Designing a DC vaccine using these antigens allows for a personalised approach to treatment. Personalised cancer vaccines can be manufactured using computer software to predict neo-antigens and generating artificial peptides that can be pulsed with DCs [121,143,144]. The main issues with generating vaccines using this method are (1) the time it takes to generate a vaccine, and (2) the cost of identifying neo-epitopes.

Incubating DCs with whole tumour lysate or killed tumour cells bypasses the need to identify neo-antigens and generate peptides [145]. Additionally, these methods allow a wide range of neo-antigens to be presented on DCs, increasing the range of antigens displayed on MHC-molecules. Tumour lysate as a source of TAA has been used to generate vaccines to successfully treat ovarian cancer [121] and breast cancer [146]. When conducting a pilot clinical trial testing a DC vaccine pulsed with oxidized autologous whole-tumour lysate administered alone, in combination with bevacizumab and low-dose cyclophosphamide, results showed an amplified T cell response against mutant neo-epitopes [121]. Interestingly, T cell exhibited a higher avidity to previously known neoepitopes but also showed priming of T cells to previously unrecognised neo-epitopes. These are neo-epitopes that could have been missed if artificially generating peptides to prime DCs.

#### 4.2.2. Dendritic Cell Maturation

The generation of vaccines requires DCs to be stimulated in vitro using a specific maturation cocktail as the cytokines used for differentiation can impact downstream T cell response. The desired outcome of maturation is to induce high expression of MHC I and MHC II molecules and upregulate the secretion of inflammatory cytokines and chemokines. Cocktails are designed to mimic the conditions of maturation in vivo, containing a mixture of pro-inflammatory cytokines or pathogen recognition receptor agonists [147]. When DC vaccines were initially trialled, the typical maturation-cocktail included pro-inflammatory cytokines, e.g., TNFα, IL-6 mixed with PGE2 (prostaglandin 2). PGE2 was used as it had been proven to promote DC migration, however it was later suggested that PGE2 may be reducing the anti-tumour immune response through inducing the differentiation of T_reg_ cells and preventing the production of some interleukins needed for DC cell maturation [148,149]. Tumour cells have the potential to utilise circulating PGE2, or create their own, to impair NK cells survival and their ability to produce chemokines which attract cDC1 cells to the tumour microenvironment, aiding tumour cells evasion of the immune system [150]. From these tests it became clear that the cocktail used for maturation has a major effect on the success or failure of DC vaccines. Later, cocktails including Toll-like receptor (TLR) agonists, co-stimulatory receptors and electroporation with mRNA encoding proteins have been tested [151,152]. Maturing DCs using a strategy known as TriMix (electroporation with mRNA encoding DC40, DC70 and TLR4) is now being explored. Co-electroporation of TriMix DCs alongside whole tumour-antigen-encoding mRNA has been reported to induce an antigen-specific T cell response in melanoma patients [152]. This method could be the future of DC maturation as incubating with cytokines can take ~24 h to produce mature DCs. However, this long incubation period can lead to DC exhaustion, reducing the immune response activated by the vaccine [152]. Tri-Mix DC vaccines have been successfully administered alongside Ipilimumab in patents with advance melanoma [153], suggesting that the effect of vaccines can be increased when combined with other immunotherapies.

#### 4.2.3. Administration of DC Vaccines

Reduced immune response may also be caused by the administration of DC vaccines. The site of injection greatly affects the migration of mature DCs to lymph organs. When treating prostate cancer using Sipuleucel-T the vaccine is administered intravenously [123]. However, in murine models this method of injection has been shown to result in DCs accumulating in vascular tissues rather than lymph nodes, failing to activate T cells for defence [154]. Migration studies show that intra-nodal injection results in the highest number of DCs reaching the lymph nodes due to no need for migration [155]. Injected DCs accumulate in the injected node before draining into the lymph nodes. As seen when treating ovarian cancers with personalised DC vaccines, this injection needs to be completed under ultrasound guidance [121].

### 4.3. Dendritic Cell Vaccines in Combination Therapy

Alongside clinical trials testing the optimum vaccine preparation methods, trials are also being conducted to test the effect of vaccines alongside other therapies (Table 3). The clinical benefit of DC vaccination when used as a monotherapy for advanced/metastasis disease is limited with only very modest increases in progression-free survival. Treatments such as chemotherapy and radiotherapy which act to reduce the size of tumours may function as an adjuvant alongside DC vaccines to help bolster anti-tumour immune responses [156]. Furthermore, an increased immune response could be seen when DC vaccines are used in combination with other immunotherapies, e.g., checkpoint inhibitors; this induced immune response can eradicate the tumour.

A phase III trial is currently testing activated autologous dendritic cell vaccines (DCVAC) in patients with relapsed ovarian cancer, fallopian tube cancer or peritoneal carcinoma in combination with platinum-based chemotherapy (NCT03905902). Patients will receive either a placebo or the vaccine alongside chemotherapy, with or without bevacizumab. The aim for this study is to determine which combination of treatments improves overall survival. This, along with other studies, should enhance the efficacy of DC-based immunotherapy in the future.

## 5. Oncolytic Viruses

**Figure 6:**
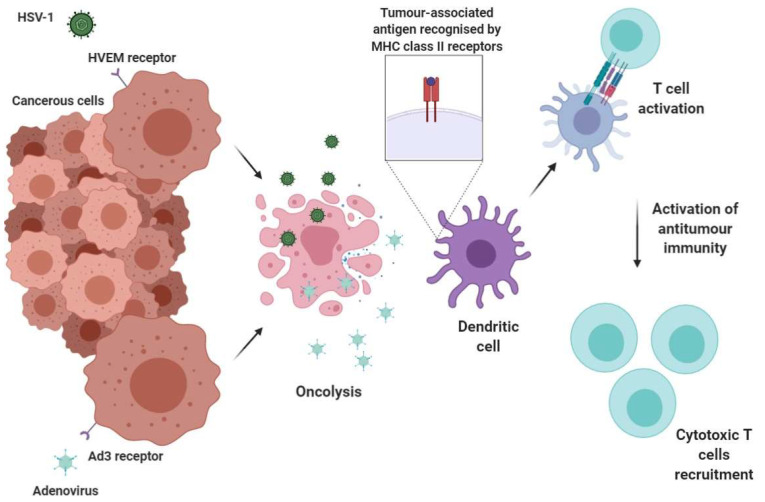
Mechanism of action of oncolytic viruses. Attachment to cancerous cells by OVs is realised through receptors found in high quantities on their surface. OVs are replication-attenuated and cannot infect normal cells due to restrictions which limit their infectivity to proliferating cells. Once the infection is established, continuous replication will finally lead to oncolysis and the spread of neoantigens in the microenvironment. Antigen-presenting cells can therefore process the tumour-associated antigens and cause activation and recruitment of cytotoxic T cells. Both oncolysis and OV mediated anti-tumour immunity are the basic mechanisms of function of these agents.

Oncolytic viruses (OVs) are viruses with a specific affinity for cancer cells that help illicit powerful anti-tumour immune responses. Depending on the type of cancer, its location, tumour microenvironment and many other factors, OVs can be manufactured to detect, infect and replicate inside cancerous cells, releasing pathogen associated molecular patterns (PAMPs) and damage associated molecular patterns (DAMPs) that can be further recognised by antigen presenting cells (APCs) and can lead to the recruitment of adaptive immune system cells (Figure 6) [157]. Therefore, the OVs work by lysing cancer cells and triggering a systemic immune response against the released tumour antigens (Figure 6).

Tumour tropism represents an important aspect in engineering these viruses and it can be either a native feature of the virus or gained through the insertion of specific genes, expressing ligands that can bind to receptors ubiquitously present in cancers such as adenovirus type 3 (Ad3) or herpes virus entry mediator (HVEM) [157,158]. Cancer cells present a unique opportunity for viruses since their antiviral responses are often defective and they highly express the enzymes and factors required for rapid cell division [157,159,160,161]. Therefore, viruses, with a natural tropism, are able to effectively infect these highly replicative cells.

Another benefit of these agents is insertion of therapeutic genes in their viral genome, so that an anti-tumour immunity can be strongly induced [162]. Nevertheless, the number of genes that can be inserted is highly determined by the size of the viral genome and the location of the targeted tumour. For instance, RNA based viruses can cross the blood brain barrier and target central nervous system tumours, if administered systemically, but can become unstable or generate low titres when a large number of genes are incorporated [157]. In contrast, viruses with larger genomes can have multiple genes, needed in the activation of the immune system inserted [163].

These features have been utilised in designing OVs as a form of therapeutic agents; some of which have already been approved by the FDA and other regulatory authorities. This development process however faces a number of challenges which are addressed by continued research in this area.

### 5.1. Development and Recent Advances

Although the concept of using viruses to destroy cancer cells arose in the 19th century, limited success and low profiling of this virotherapy strategy meant that very little research was conducted. However, it was the discovery of genetic engineering that brought about a renewal of interest in virotherapy that allowed the generation of more potent, tumour-specific oncolytics. This initiated active research in the discovery of safer, more selective virotherapies, such as herpes simplex virus (HSV) and adeno virus-based therapies in cancer, leading to successful clinical trials and approvals by health regulatory bodies worldwide.

#### 5.1.1. Herpes Simplex Virus Type 1 (HSV-1)

HSV-1 is a large double-stranded DNA pathogen, known for its neurotropism and the capability of causing lifelong infection [164]. Its tumoricidal potential was considered following the deletion of thymidine-kinase (*dl*sptk), a gene involved in the regulation of thymidine levels and is crucial for virus replication [165]. The removal of this essential gene rendered a replication-attenuated virus reliant on proliferating cells (e.g., cancer cells) for their successful replication [164]. The innate neurotropism and the conditional replication achieved through *dl*sptk established perfect features for targeting brain cancers, but its advancement into clinical trials was stopped due to the resistance to antiviral drugs gained through *dl*sptk deletion and undesirable toxicities at high titres [166]. Since *dl*sptk modifications have been implemented through genetic engineering to lower neurotoxicity of HSV-1 based OVs while still maintaining their ability to target actively dividing cells. Deletion of both $\gamma_{1}34.5$ g genes (blocks shut-off of protein synthesis in the infected cells to induce neurovirulence) and disruption of $U_{L}39$ (allows viral growth in non-dividing cells) improved the susceptibility of these OVs to acyclovir and ganciclovir, restricted its replication to actively dividing cells and prevented latency establishment [164,166]. G207, an improved HSV-1 oncolytic, possesses these improvements and is currently in a phase I clinical trial (NCT02457845) for the treatment of recurrent supratentorial brain tumours in children. In previous clinical trials, G207 demonstrated a lack of toxicity, anti-tumour activity and median survival of 7.5 months in patients suffering from recurrent malignant glioma [164]. An altered version of G207, G47Δ included deletion of the α47 gene enabling the virus to be more effective in producing oncolysis and improving tumour associated antigen presentation [167]. G47Δ is currently under clinical investigation (JPRN-UMIN000002661) in glioblastoma. Another similar oncolytic HSV-1 is HSV1716, which has the $U_{L}39$ gene but the activity of $\gamma_{1}34.5$ genes was lost through insertion of mutations within their sequences [164]. HSV1716 was tested in third phase I clinical trial in high-grade glioma and reported absence of toxicity and increased survival (15–22 months) in 25% of the patients [168].

Improvements in initiating anti-tumour effects were achieved through the insertion of the therapeutic gene *interleukin-12* (*IL-12*). M032 is a second-generation OV that bears the *IL-12* gene, inducing T cells immunologic effects in murine models and is currently tested in a phase I clinical trial (NCT02062827) [169].

More recently, in October 2015, FDA and EMA approved the use of Talimogene Iaherparepvec (T-VEC) in the treatment of advanced melanoma [170]. T-VEC is an attenuated HSV-1 engineered that expresses human granulocyte-macrophage colony- stimulating factor (GM-CSF), which is a regulatory cytokine that functions in the recruitment and maturation of antigen-presenting cells [157,170]. Apart from exhibiting immune-mediated regression of distant lesions, the intratumorally administration of the virus avoids serum neutralization, which poses a technical challenge depending on the tumour location [171,172]. Nevertheless, the overall survival rate reported was 23.3 months in 26% of the patients, as well as 15% of visceral metastasis reduction [173]. However, one significant issue that has been encountered in the utilisation of this agent is the lack of training of the oncologists in safe handling, preparation and administration of T-VEC in patients [170].

#### 5.1.2. Adenovirus

This family of double-stranded DNA viruses causes upper-respiratory tract and gastrointestinal infections in humans [157]. However, their potential in targeting tumours has been observed following an induction of decreased pathogenicity and replication conditioning through mutations. For instance, the early region 1B (E1B) gene deletion in a modified human adenovirus serotype 5 (ONYX-015) is a strategy for attenuating the pathogenicity of the virus, as it protects the infected cells from the E1A-induced p53 effects [157,174]. The lack of E1B dictates the tumour tropism feature, limiting infectivity to cells that provide RNA export, which is not found in normal cells [175]. A completed phase I trial utilised ONYX-015 in the treatment of recurrent malignant glioma with a good outcome (median survival time 6.2 months) with no adverse effects observed and no maximum tolerated dose reported [176]. The effectiveness of adenovirus serotype 5 in oncolytic virotherapy was further confirmed by the approval of Oncorine (H101) in conjunction with chemotherapy by the Chinese SFDA in November 2005 in the treatment of HNSCC or oesophageal squamous cell carcinoma [164,177]. Clinical studies performed on a multicentre, randomized and controlled phase III trial showed a significantly higher response rate in the Oncorine plus chemotherapy-treated HNSCC patients (78.8%), compared with the chemotherapy treated control SCCHN patients (39.6) [177]. No serious adverse events were detected apart from fever, local site pain and flu like symptoms [177]. Following the completion of the study, the oncolytic virus was approved for use in China. The same company, Shanghai Sunway Biotech Co., Ltd., is currently developing other two adenoviruses: H103 and H102, with the former one in phase II clinical trial and the latter still in preclinical stage.

Another genetically engineered oncolytic virus, DNX2401, uses the genome of an adenovirus-gamma 24 with a cyclic arginine/glycine/aspartic acid (RGD-4C) peptide motif inserted in it and a Δ-24 mutation [178]. DNX2401 preference of infecting cancerous cells was achieved through the addition of RGD-4C, as it permits attachment to integrins, which are ubiquitously present on glioma cells, and overcomes the relative low expression of CARs (coxsakie-adenovirus receptors) [178]. The Delta-24 mutation restricts viral replication to cells defective in the retinoblastoma protein tumour suppressor (RB1), allowing DNX2401 to selectively replicate in tumour cells that have lost RB1 [178]. The latest data from an ongoing phase I study (NCT00805376) suggests dramatic responses with long-term survival in recurrent high-grade gliomas and more than 95% in tumour size [179]. Infiltration of T cells with Th1 immune response was observed, as well as an adaptive immune memory effect 2.5 years after a complete response [179].

Currently there are numerous virotherapies that are being tested in clinics and the most promising ones are focused on brain cancer. Central nervous system (CNS) malignancies usually show a dim prognosis with a median survival rate of just 14.6 months in the case of glioblastoma according to WHO [180]. The interest in virotherapy for this type of cancer has risen in the last decade mainly due to the ability of RNA-based OVs to cross the blood-brain barrier when administered systemically, and generally no maximum tolerated doses reported [180].

When compared with other immunotherapies OVs seem to be more potent anti-tumour immunity inducers [181]. The issues regarding immunosuppressive tumour microenvironment, often encountered by T cells therapies when treating solid malignancies is overcome by OVs [31,181]. It has been known that oncolysis of cancerous cells lead to a rapid maturation and antigen presentation by BAFT3+ DCs, therefore, recruitment of T cells at the site of infection and their activation is promoted [157]. Moreover, some groups have demonstrated that the tumour microenvironment immunosuppression is reversed by OVs, and due to cytokine signalling, the infiltration of immune cells within the tumour is increased [171]. Interestingly, the ability of initiating anti-tumour immune responses differs in OVs, one study on murine models demonstrating that adenovirus based OVs would be better at enabling T cell anti-tumour activity than vaccinia virus, HSV and reovirus based OVs [181]. Nevertheless, antiviral immunity has to be considered when designing OVs with strong immunity activation, as virus clearance can occur faster than tumour-associated antigen presentation process [157].

#### 5.1.3. Combination Therapy- Pre-Clinical and Clinical Trials

**Figure 7:**
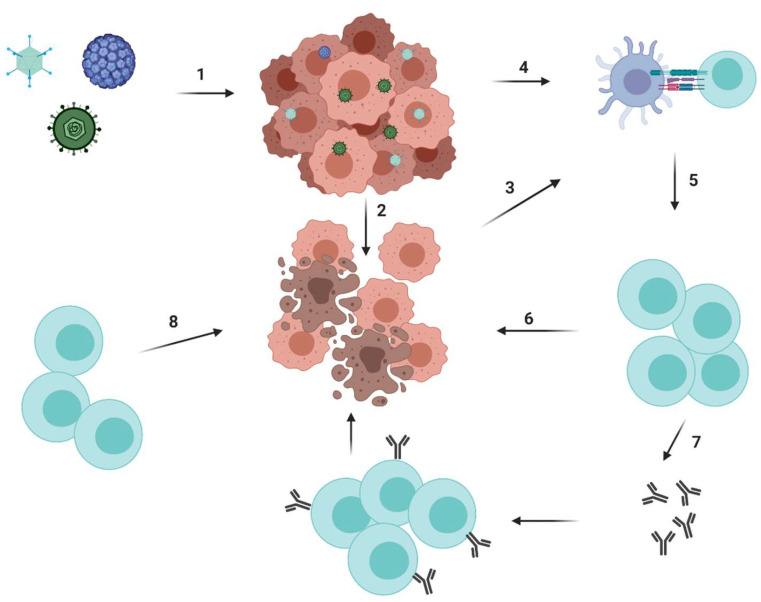
Enhancement of tumouricidal effect through combination immunotherapy. OVs infection of cancerous cells (**1**) leads to oncolysis (**2**) and reverse the immunosuppression of tumour microenvironment. DAMPs and PAMs released by bursting cells are recognised by DCs and presented to T cells (**3**). Virus progenies or viral components can be detected as well by DCs (**4**). Initiation of an anti-tumour immune response by DCs (**5**) needs to happen before activation of antiviral immunity and most OVs are designed to delay their detection. The anti-tumour immune cells are attracted by cytokines towards the tumour (**6**). The response against the tumour is enhanced by immune checkpoint inhibitors (**7**), as downregulation of cytotoxic T cells by Tregs or cancer itself is inhibited. CAR-T cells can enter the tumour more easily due to reverse immunosuppression of the tumour microenvironment (**8**).

Altering the tumour microenvironment is the most attractive feature for combination strategies with other immunotherapies such as checkpoint inhibitors, dendritic vaccines and CAR T-cells, where the entrance problem is often encountered (Figure 7). Combination therapy of already approved agents in the treatment of cancer and their synergistic effect on the overall survival rate in patients are being widely explored at the moment.

Melanoma is susceptible to immunotherapies and targeting it with both OVs and immune checkpoint inhibitors that were specifically designed to target this cancer and have previously been approved for clinical use was thought to have a better outcome, compared with monotherapies [182]. However, the data obtained from a phase II clinical study (NCT01740297) on patients with unresectable stage IIIB-IVM1c malignant melanoma suggests that pseudo-progression (a delayed tumour shrinkage following an increase in tumour burden after treatment) is common in immune checkpoint inhibitor therapies, T-VEC and T-VEC plus ipilimumab [183]. The lower pseudo-progression incidence was reported in checkpoint inhibitor monotherapy, and the duration response (DOR) was longer for patients without pseudo progression versus those with pseudo progression [183]. Furthermore, 39% of patients had an objective response and regression of visceral lesions was observed in 52% of patients [184].

In preclinical assays made on murine models, many OVs have shown better results in the overall survival rate and cancerous cell destruction when combined with immune checkpoint inhibitors [185]. The insertion of immunomodulatory cytokine genes within the oncolytic virus genome, however, proved to be even more efficacious at inducing anti-tumour immunity in the case of acute myeloid leukemia (AML) and colon cancer in mice [186,187]. Another preclinical study suggests that the combination of an oncolytic adenovirus with immune checkpoint inhibitors can broaden the spectrum of T-cell responses and reverse the systemic resistance to PD-1 immunotherapy [185].

The potential of combining checkpoint inhibitors with OV therapy is currently being tested in a phase II clinical study (NCT02798406) where the adenovirus DNX-2401 with pembrolizumab are used in the treatment of glioblastoma (GBM) and gliosarcoma. Interim results of this phase II revealed that there was no dose-limiting toxicity or unexpected safety issues with 47% of the patients experienced clinical benefit and two patients with >94% tumour regression [188]. Following the evaluation of data obtained from the phase I trial (NCT00805376), avoidance of tumour immune suppression through checkpoint inhibitors has been strongly implicated as a way to help augment clinical benefit [179]. T-cell exhaustion was partially overcome by DNX-2401 in NCT00805376 as its administration induced a decrease in transmembrane immunoglobulin mucin-3 (TIM-3) (discussed in Section 3.2.2) [179]. As T-cell exhaustion is often a result of the tumours’ ability to supress immunity, inhibiting these receptor/ligand axis has been shown to reverse this mechanism in advanced melanoma patients and the combination therapy used in this phase II trial (NCT02798406) might have a great success [189]. However, pseudo-progression was observed as well during the tumour regression period in the phase I trial and it should be considered in phase II study, given the fact that it has been seen in T-VEC and ipilimumab trials as well. Other combination clinical trials using DNX-2401 include a phase I trial in combination with temozolomide (NCT01956734) for the treatment of GBM and a phase Ib trial for the treatment of GBM or gliosarcoma (NCT02197169) that uses DNX-2401 alone or in combination with interferon-γ [164,190].

## 6. Future Directions

Advances in immunotherapies against both solid tumours and haematological malignancies have shown promise and an increase in clinical benefits in recent years. However, the complex dynamic of the TME, the heterogeneity of tumours and their histological features pose everlasting challenges in achieving improved treatment efficacy. Despite the successes in targeting non-tumour components, including immunosuppressive mechanisms, our lack of understanding of the cellular and molecular crosstalk in the tumour niche results in only a few patients exhibiting objective control of tumour progression. Therefore, numerous investigations are being carried out to identify potential targets such as galectin-1, cancer associated cathepsins, OX40+Foxp3+ T_reg_ cells, etc., to develop new therapies and to achieve greater treatment efficacy. Such recent and ongoing efforts are discussed below.

### 6.1. Galectin-1 and Its Tumour-Immune Suppressing Role

Galectin-1 (Gal1) is a key pro-tumourigenic player with multiple roles in the TME. This glycoprotein is secreted by tumour cells and induces tumour cell proliferation, migration and invasion of local tissue, reviewed elsewhere [191]. Tumour-secreted dimeric Gal1 binds to extracellular matrix proteins such as fibronectin, collagen and laminin, and cell-surface glycoconjugates mediating a bivalent cross-linking between tumour cells and the stroma, inducing homotypic tumour cell aggregation, invasion and metastasis [191]. Interestingly, in the later stage of tumour development, tumour cells, tumour-associated macrophages and T cells, and local microvascular endothelial cells overexpress and secrete Gal1, resulting in further tumour growth and angiogenesis via both autocrine and paracrine interactions [191,192,193,194,195,196,197]. However, the poor prognosis associated with this elevated level of Gal1 is now thought to be primarily related to its immunosuppressive mechanisms [198].

Earlier studies from both in vitro and in vivo experiments demonstrated an increase in extracellular Gal1-induced apoptosis in Th1 cells via its interaction with CD43, CD45 and CD7 receptors, resulting in tumour-induced immune evasion [199,200]. More recently, it was reported that extracellular Gal1 induces a switch from a Th1 and Th17 cytokine secretory profile to Th2 response (IL5, IL-10 and TGF-beta) [201,202,203,204]. Th2-mediated cytokine response is more anti-inflammatory, and it was reported that Th2-secreted Gal1 selectively induces apoptosis of Th1 and promote Th2 cytokine secretion [205]. Consistent with this, it was found that Gal1-deficient mice had enhanced Th1 and Th17-mediated immune response, indicating a potent tumour-induced immune evasion role of Gal1 in the glioma TME [206].

It was thought that Gal1 secreted from large tumours induces T cell apoptosis and switches to Th2 cytokine production. However, recent data suggests that Gal1 conditions the tumour endothelial cells by inducing expressions of PD-L1 and Gal9 and suppresses anti-tumour immunity by causing T cell exclusion, which in fact takes place relatively earlier during tumour growth [207]. Thus, any lack of successes in anti-PD-1 treatment, whereby patients eventually develop progressive disease, may be due to Gal1-induced immune-evasion and addressed by introducing anti-Gal1 antibody in combination with anti-PD-1 treatment. Indeed, Gal1 blockade enhanced the effect of anti-PD-1 therapy in preclinical models of head and neck cancers [207]. Therefore, this strategy of blocking Gal1 in combination with anti-cancer immunotherapies should be investigated further and introduced in clinical studies.

Another evolving anticancer treatment is a combination of radiation therapy and anti-PD-1 therapy. Early clinical data (phase I) showed an improved outcome of the combination of photon radiation with anti-PD-1 immunotherapies in patients with non-squamous non-small cell lung carcinoma (NSCLC) [208], which has moved onto phase II (NCT03044626). Interestingly, radiotherapy results in an elevated extracellular Gal1 level [209] and, therefore, patients undergoing this combination therapy may be at a disadvantage as secreted Gal1 can induce T cell exclusion via PD-L1 and Gal9 expression. Thus, introducing Gal1-blockade into the combination strategy may improve efficacy of this anticancer treatment. Indeed, in preclinical models with Gal1-KO tumours, this combination (radiation and anti-PD-1 treatment) showed a greater anti-tumour response, with no detectable tumour in 70% of the mice bearing HNC post-radiotherapy initiation [207]. Therefore, therapeutically targeting this potent pro-tumourigenic and immunosuppressing factor in combination with other anti-cancer therapies may be vastly clinically beneficial for a number cancers [210].

### 6.2. Cathepsins in Cancer

Cathepsins are lysosomal enzymes which are highly expressed in tumour cells in response to the hypoxic and slightly acid microenvironment. A number of these cathepsin enzymes has been implicated in the progression of tumour growth and metastasis in the last two decades [211,212,213]. Notably, aspartyl cathepsin D gained an increased attention due to their extracellular presence in the TME [211,214]. Cathepsin D has been shown to be over expressed and hyper-secreted in a number of cancers including breast, ovarian, lung, prostate and malignant glioma cancers, and known as a marker for poor prognosis in breast cancer patients [211,215]. Over the last three decades, extracellular cathepsin D has been reported to play a pro-tumourigenic role, inducing tumour growth, invasion and angiogenesis. This is particularly true for triple negative breast cancer (TNBC), which accounts for 15–20% of all breast cancers, with limited treatment options [216,217]. In this context, patients with TNBC have an unfavourable prognosis besides high risk of metastases, increased risk of tumour relapse, and worse survival rate, compared with other breast cancer subtypes. There is ample of evidence which demonstrates that high levels of CathD in TNBC primary tumours is indicative of local recurrence or distant metastasis [218,219]. Furthermore, CathD expression is suggested to be an independent prognostic factor for disease-free survival of TNBC patients [219]. Therefore, targeting extracellular CathD immunotherapeutically may reduce tumour growth and subsequent metastasis. To address this, Ashraf et al. recently demonstrated an anti-tumour efficacy for anti-CathD antibody in triple-negative breast cancer (TNBC) mice models [220]. The two human anti-CathD Abs used in this study efficiently bound to human and mouse CathD resulted in a significant inhibition of tumour growth in three different TNBC mouse models (MDA-MB-231 cell xenografts and two TNBC patient-derived xenografts) [220]. Furthermore, the recruitment of the immune-suppressing tumour-associated macrophages (TAMs) and myeloid-derived suppressor cells within the TME were effectively blocked. This preclinical study demonstrated a promising proof-of-concept that may also be utilised in peritoneal metastases, such as high-grade serous carcinoma (advanced ovarian cancer), where TAMs constitute over 50% of cells and ascites [221]. More recently, co-expression of androgen receptor with CathD in TNBC patients showed poorer overall survival, demanding the use of anti-CathD targeting in combination therapy [222]. Therefore, targeting cathepsins in cancers utilizing an immunotherapy approach in combination with conventional chemotherapy and/or nanoparticle-based intervention [223,224,225] may be more efficacious. However, bioavailability, selective targeting, and drug-delivery routes pose greater challenges which would require further research.

### 6.3. OX40-Positive Regulatory T Cells and Plasmacytoid DCs

Although they are indispensable at preventing autoimmunity, Foxp3-expressing T_regs_ has been known to suppress effective anti-tumour immunity [226]. T_regs_ infiltrate into tumour hosting tissues, which is often associated with poor prognosis in cancer patients [227,228,229]. Therefore, removing these cells or reducing their activity in the TME, without negatively impacting its anti-autoimmunity role, is a key focus in improving immunotherapeutic approaches.

A number of strategies have been tested in an attempt to dampen down the immunosuppressive effects of T_regs_ in TME (reviewed in [3]), such as T_regs_ depletion using anti-CD25 mAbs, targeting immune checkpoint proteins (e.g., OX40 [230]), selectively targeting cell signalling (e.g., PI3Kδ) in T_regs_ (phase I, NCT02646748) [231], etc. Amongst these, targeting OX40, a member of the tumour necrosis factor receptor (TNFR) family [232], has shown promising anti-tumour activity. OX40 is constitutively expressed on FoxP3+ T_regs_ that under certain conditions can inhibit the generation of Foxp3 T_regs_ [233]. Several anti-OX40 agonistic mAbs are currently being tested, either alone or in combination with other immunotherapies, in early phase cancer clinical trials [234]. A phase I trial (NCT01644968) analysed the toxicity of the intravenous administration of three doses of 9B12 murine agonistic anti-human OX40 mAb in advanced stage solid cancer (melanoma, renal, urethral, and prostate cancers) patients (*n* = 30), with 12 patients having regression of at least one metastatic lesion after a single course of treatment [234]. Another OX40 targeted therapies that showed promise in patients previously treated with immunotherapy is the human anti-OX40 agonistic mAbs (PF-04518600) which resulted in a stable disease for more than six months with increased number of memory T cells [235]. Currently, a combination of MEDI6383, an OX40 ligand-Fc fusion protein, and anti-PD-L1 drug durvalumab is being investigated in patients with selected advanced stage solid tumour with the preclinical evidence that this combination has significantly expanded and increased the effector characteristics of mature T cells in the tumour-draining lymph nodes and tumour itself [236,237]. This dual therapy also resulted in an increase in CD8+/T_reg_ ratio, leading to rapid tumour shrinkage [236,237].

More recently, OX40-positive plasmacytoid DCs (major producers of IFN-I) were discovered to promote anti-tumour immunity in the TME [238]. pDCs are major producers of antiviral type I interferon (IFN-I). However, recently it was reported that a subset of naturally occurring OX40+pDCs increase local IL-12 and IFN-α production and enhances anti-tumour conventional DCs (lymphoid-tissue resident) and CD8+ T cells interactions in vivo in the TME of head and neck carcinomas [238]. Interestingly, Lu et al. reported a key anti-tumour role for IFN-I in enhancing cytotoxic T cell effector function in suppressing tumour development [239], which may coincide with the effect of the recently discovered OX40+pDCs. Thus, inducing upregulation of OX40 in intratumoural pDCs as well as Foxp3+ T_regs_ in combination with other anti-cancer treatments may enhance anti-tumour immune responses and clinical benefit.

## 7. Conclusions

Anti-cancer immunotherapy as monotherapy or in combination has shown promising results in several cancers. Although earlier successes were more evident in haematological malignancies, more recent reports show increased clinical benefits in patients with solid tumours; in particular, oncolytic virotherapy, which is able to cross the blood brain barrier and kill cancer cells directly but also indirectly by enhancing T cell trafficking in the TME. However, resistance to therapies via mechanisms such as tumour-induced immune evasion, may lessen the therapeutic efficacy. Therefore, further research efforts should focus on rational combinations of immunotherapies to help overcome the anti-tumour immune resistance and help drive clinical benefit.

## Figures and Tables

**Table 1:** Current clinical trials for CAR T cells targeting solid tumours.

Target	CAR Construct	Malignancy	Phase	Reference Clinicaltrials.gov
**CD70**	CD3ζ, CD28	Pancreatic, renal cell, breast, melanoma and ovarian	I and II	NCT02830724
**Mesothelin**	CD3ζ, 4-1BB	Pancreatic, ovarian and mesothelioma	I	NCT02159716
**Muc16 (CA125)**	CD3ζ, CD28 Armoured with IL-12 secretion	Ovarian	I	NCT02498912
**HER2**	CD3ζ, CD28	Glioblastoma	I	NCT02442297
**Glypican-3**	CD3ζ, CD28 and 4-1BB	Hepatocellular carcinoma	I	NCT02395250
**CEA**	CD3ζ, CD28	Peritoneal carcinomatosis, colorectal, gastric, breast and pancreatic cancer	I	NCT03682744
**EGRFvIII**	CD3ζ, 4-1BB	Glioblastoma	I	NCT03726515
**PSMA**	CD3ζ, CD28	Prostate Cancer	I	NCT01140373

Abbreviations: HER2, Human Epidermal growth factor Receptor 2; CEA, Carcinoembryonic Antigen; EGFRvIII, Epidermal Growth Factor Receptor variant III; PSMA, Prostate Specific Membrane Antigen.

**Table 2:** Approved anti-PD-1/PD-L1 therapies in the clinics.

Drug	First FDA Approval Date	Cancer Type	Ref
Pembrolizumab (Anti-PD-1)	2014	Melanoma; non-small cell lung cancer; head and neck squamous cell cancer; classical Hodgkin lymphoma; primary mediastinal large b-cell lymphoma; urothelial carcinoma; microsatellite instability-high cancer; gastric cancer; cervical cancer; hepatocellular carcinoma; merkel cell carcinoma	[73]
Nivolumab (Anti-PD-1)	2014	Unresectable or metastatic melanoma; adjuvant treatment of melanoma; metastatic non-small cell lung cancer; small cell lung cancer; advanced renal cell carcinoma; classical Hodgkin lymphoma; squamous cell carcinoma of the head and neck; urothelial carcinoma; microsatellite instability-high (MSI-H) or mismatch repair; deficient (dMMR) metastatic colorectal cancer; hepatocellular carcinoma	[74]
Cemiplimab (Anti-PD-1)	2018	Metastatic cutaneous squamous cell carcinoma or locally advanced cutaneous squamous cell carcinoma who are not candidates for curative surgery or curative radiation	[75]
Atezolizumab (Anti-PD-L1)	2016	Locally advanced or metastatic urothelial carcinoma; metastatic non-small cell lung cancer; locally advanced or metastatic triple-negative breast cancer	[76]
Durvalumab (Anti-PD-L1)	2017	Locally advanced or metastatic urothelial carcinoma	[77]
Avelumab (Anti-PD-L1)	2017	Metastatic Merkel cell carcinoma (>12 yo); Locally advanced or metastatic urothelial carcinoma; advanced renal cell carcinoma	[78]

**Table 3:** Selective ongoing clinical trials for DC vaccines.

Start Date	Title	Conditions	NCT Number	Intervention	Phase	Current Status
2012	Natural Dendritic Cell Vaccines in Metastatic Melanoma Patients	Melanoma	NCT01690377	Biological: PDC or myDC	I	Complete
2013	Safety Study of a Dendritic Cell-based Cancer Vaccine in Melanoma (GeniusVac-Mel4)	Melanoma	NCT01863108	Biological: GeniusVac-Mel4	I	Complete
2015	myDC/pDC in Stage III Melanoma Patients	Melanoma	NCT02574377	Drug A: myDC vaccination Drug B: pDC vaccination Drug C: combined myDC/pDC vaccination	I & II	Unknown
2016	Melanoma Patients Immunized with Natural Dendritic Cells (MIND-DC)	Melanoma	NCT02993315	Biological: nDC vaccination Biological: Placebo injection	III	Active, not recruiting
2016	Natural Dendritic Cells for Immunotherapy of Chemo-naive Metastatic Castration-resistant Prostate Cancer Patients	Prostatic Neoplasms	NCT02692976	Biological:mDC vaccination Biological: pDC vaccination Biological: mDC and pDC vaccination	II	Complete
2017	Dendritic Cell Therapy, Cryosurgery, and Pembrolizumab in Treating Patients With Non-Hodgkin Lymphoma	Non-Hodgkins Lymphoma	NCT03035331	Procedure: Cryosurgery Biological: Dendritic Cell Therapy Biological: Pembrolizumab Biological: Pneumococcal 13-valent Conjugate Vaccine Other: Quality-of-Life Assessment	I & II	Recruiting
2018	Intratumoral Injection of Autologous CD1c (BDCA-1)+ Myeloid Dendritic Cells Plus Talimogene Laherparepvec (T-VEC) (myDCTV)	Melanoma	NCT03747744	Other: CD1c (BDCA-1)+ myDC	I	Recruiting
2018	Intratumoral Injection of Autologous CD1c (BDCA-1)+ myDC, Avelumab, and Ipilimumab Plus Systemic Nivolumab (myDAvIpNi)	Solid Tumours Metastases of Soft Tissue	NCT03707808	Drug: intratumoral injection of autologous CD1c (BDCA-1)+ myDC	I	Recruiting
2019	Treatment of Recurrent Bladder Cancer With Dendritic Cells	Bladder cancer	NCT04184232	Biological: Dendritic cells Other: Standard treatment according to the Clinical protocols	II	Recruiting
2019	Dendritic Cells for Immunotherapy of Metastatic Endometrial Cancer Patients (DECENDO)	Endometrical Cancer	NCT04212377	Biological: Dendritic Cells for endometrial cancer	II	Recruiting
2019	Arm 1: Infusion of Autologous Monocyte-derived Lysate Pulsed Dendritic Cells (PV-001-DC) in Patients With Advanced Melanoma	Metastatic Melanoma	NCT03803397	Biological: PV-001-DC	I	Not yet recruiting
2019	DCVAC/OvCa and Standard of Care (SoC) in Relapsed Ovarian, Fallopian Tube, and Primary Peritoneal Carcinoma (VITALIA)	Ovarian Cancer Fallopian Tube Cancer Peritoneal Carcinoma	NCT03905902	Biological: DCVAC/OvCa Bioloigcal: DCVAC/OvCa placebo	III	Not yet recruiting
